# Characterization of hemicellulase and cellulase from the extremely thermophilic bacterium *Caldicellulosiruptor owensensis* and their potential application for bioconversion of lignocellulosic biomass without pretreatment

**DOI:** 10.1186/s13068-015-0313-0

**Published:** 2015-08-28

**Authors:** Xiaowei Peng, Weibo Qiao, Shuofu Mi, Xiaojing Jia, Hong Su, Yejun Han

**Affiliations:** National Key Laboratory of Biochemical Engineering, Institute of Process Engineering, Chinese Academy of Sciences, Beijing, China

**Keywords:** Lignocellulose, *Caldicellulosiruptor*, Thermophilic enzyme, Synergetic hydrolysis, Pretreatment, Cellulase, Hemicellulase

## Abstract

**Background:**

Pretreatment is currently the common approach for improving the efficiency of enzymatic hydrolysis on lignocellulose. However, the pretreatment process is expensive and will produce inhibitors such as furan derivatives and phenol derivatives. If the lignocellulosic biomass can efficiently be saccharified by enzymolysis without pretreatment, the bioconversion process would be simplified. The genus *Caldicellulosiruptor*, an obligatory anaerobic and extreme thermophile can produce a diverse set of glycoside hydrolases (GHs) for deconstruction of lignocellulosic biomass. It gives potential opportunities for improving the efficiency of converting native lignocellulosic biomass to fermentable sugars.

**Results:**

Both of the extracellular (extra-) and intracellular (intra-) enzymes of *C. owensensis* cultivated on corncob xylan or xylose had cellulase (including endoglucanase, cellobiohydrolase and β-glucosidase) and hemicellulase (including xylanase, xylosidase, arabinofuranosidase and acetyl xylan esterase) activities. The enzymes of *C. owensensis* had high ability for degrading hemicellulose of native corn stover and corncob with the conversion rates of xylan 16.7 % and araban 60.0 %. Moreover, they had remarkable synergetic function with the commercial enzyme cocktail Cellic CTec2 (Novoyzmes). When the native corn stover and corncob were respectively, sequentially hydrolyzed by the extra-enzymes of *C. owensensis* and CTec2, the glucan conversion rates were 31.2 and 37.9 %,which were 1.7- and 1.9-fold of each control (hydrolyzed by CTec2 alone), whereas the glucan conversion rates of the steam-exploded corn stover and corncob hydrolyzed by CTec2 alone on the same loading rate were 38.2 and 39.6 %, respectively. These results show that hydrolysis by the extra-enzyme of *C. owensensis* made almost the same contribution as steam-exploded pretreatment on degradation of native lignocellulosic biomass. A new process for saccharification of lignocellulosic biomass by sequential hydrolysis is demonstrated in the present research, namely hyperthermal enzymolysis (70–80 °C) by enzymes of *C. owensensis* followed with mesothermal enzymolysis (50–55 °C) by commercial cellulase. This process has the advantages of no sugar loss, few inhibitors generation and consolidated with sterilization.

**Conclusions:**

The enzymes of *C. owensensis* demonstrated an enhanced ability to degrade the hemicellulose of native lignocellulose. The pretreatment and detoxification steps may be removed from the bioconversion process of the lignocellulosic biomass by using the enzymes from *C. owensensis*.

**Electronic supplementary material:**

The online version of this article (doi:10.1186/s13068-015-0313-0) contains supplementary material, which is available to authorized users.

## Background

Production of biofuels from the renewable lignocellulosic biomass is gradually considered as a promising way to replacement of fossil fuels. However, its bioconversion has been limited by its hydrolysis because the main components of the lignocellulosic biomass (cellulose, hemicellulose and lignin) are tightly held together and form lignin-carbohydrate complexes (LCC). The lignin-carbohydrate complexes create a barrier for microbial conversion [1]. Conversion of lignocellulosic biomass to fermentable sugars represents a major challenge in global efforts to utilize renewable resources in place of fossil fuels to meet the rising energy demands [2]. Enzymatic hydrolysis is the most common process to degrade the cellulose and hemicellulose into fermentable sugars such as glucose and xylose.

Currently, the high cost of enzymolysis is a major obstacle for production of biofuels at an industrial scale [3]. Exploring the highly efficient cellulase and hemicellulase is attached much attention for reducing the cost of biofuels production. Before enzymatic hydrolysis, pretreatment process is required to break down the rigid association of lignocelluloses, so that the enzymes can easily access the cellulose to hydrolyze into monomers [4]. Pretreatment, such as steam-explosion pretreatment, hydrothermal pretreatment, and acid or alkali pretreatment, allows to change the structure of the lignocellulose, such as increasing the surface area and porosity of biomass, partially removing the hemicelluloses and lignin, and reducing the crystallinity of cellulose [5]. Although pretreatment is efficient for improving the enzymatic hydrolysis of lignocelluloses, it has been viewed as one of the most expensive processing steps in biomass-to-biofuel conversion [6]. Moreover, during pretreatment process some sugars are damaged and converted into furan derivatives (furfural and HMF) and carboxylic acids, which, together with phenol derivatives (from lignin), will inhibit the fermentation process [7].

Therefore, after pretreatment the detoxification step is essential for improving the fermentation efficiency. The methods of detoxification can be divided into three main groups: biological, physical and chemical [8], such as using microorganisms or enzymes to change the inhibitors’ chemical structures [9, 10], adsorbing the inhibitors by using activated charcoal [11] and ion exchange resins [12], and adding reductive substances [13] or pH modification [14, 15]. The detoxification process is also costly and many of the detoxification methods result in sugar losses [8]. Can the pretreatment and detoxification be removed from the bioconversion process of lignocellulosic biomass?

Many extreme thermophiles are able to utilize a variety of carbohydrates pertinent to the conversion of lignocellulosic biomass to biofuels. Characterization of the enzymes from these extremely thermophilic bacteria is likely to generate new opportunities for the use of renewable resources as biofuels [16, 17]. Among them, the genus *Caldicellulosiruptor*, an obligatory anaerobic and extreme thermophile has recently attracted high interest for it can produce a diverse set of glycoside hydrolases (GHs) for deconstruction of lignocellulosic biomass [18–20]. It was reported [19] that the open *Caldicellulosiruptor* pangenome encoded 106 glycoside hydrolases (GHs) from 43 GH families. The gene clusters that encode multidomain cellulases or hemicellulases were found in the genome of *Caldicellulosiruptor*. Many novel heat-stable extracellular enzymes for biomass degradation had been heterogeneously expressed [21–26]. Especially, some enzymes are not only multimodular, but possess catalytic domains with different activities (multifunctional) [19, 27]. They differ from the two general cellulolytic enzymes systems: one with free cellulases and hemicellulases produced by fungi and most bacteria [28], and the other in which glycosidases self-assemble onto a common protein scaffold to form large macromolecular assemblies called cellulosomes [29, 30]. For example, the cellulase CelA produced from *C. bescii*, comprises a GH 9 and a GH 48 catalytic domain, could hydrolyze the microcrystalline cellulose not only from the surface as common cellulases done but also by excavating extensive cavities into the surface of the substrate [31]. The major commercial cellulolytic enzymes are currently produced by fungi with free noncomplexed cellulases and hemicellulases [32]. The cellulolytic enzymes from genus *Caldicellulosiruptor* with different characteristics may be complementary with fungal cellulolytic enzymes on hydrolysis of lignocellulosic biomass, therefore showing potential commercial application value. *C. owensensis* grows on a wide variety of carbon sources including pentose, hexose, oligosaccharide, and polysaccharide [33]. Comparing of the growth of the seven species of *Caldicellulosiruptor* (*C. bescii*, *C. hydrothermalis*, *C. kristjanssonii*, *C. kronotskyensis*, *C. lactoaceticus*, *C. saccharolyticus*, *C. owensensis*) on xylose, the cell density of *C. owensensis* was only slightly lower than *C. saccharolyticus* and higher than the other five species [18]. Moreover, the results of analyzing of the diversity of biomass deconstruction-related glycoside hydrolases in *Caldicellulosiruptor* showed that *C. owensensis* owned abundant xylan deconstruction-related glycoside hydrolases including the 5, 10, 11, 39, 43, 51 and 67 GH families, which were the total xylan deconstruction-related glycoside hydrolase families in *Caldicellulosiruptor* [18]. The diversity of the xylan deconstruction-related glycoside hydrolases and the physiological characteristics of *C. owensensis* showed that it is a promising candidate for hemicelluloses deconstruction.

The hemicellulose is much easier to be enzymatically hydrolyzed than cellulose in the native lignocellulose because the hemicellulose is amorphous. Removing hemicellulose can increase the surface area and porosity of lignocellulose hence improving the access for cellulase to touch with cellulose. The two-step hydrolysis by first using lignocellulolytic enzymes focusing on hemicelluloses deconstruction then hydrolysis by cellulase may be an efficient strategy for avoiding the current pretreatment process for biofuels production. Here we hope that the enzymes of *C. owensensis* which has the high ability of deconstructing native hemicellulose would be competent to support the two-step hydrolysis strategy.

In this work, the characteristics of hemicellulase and cellulase of *C. owensensis* cultivated on different carbon sources were assayed. The extra-enzymes and intra-enzymes of *C. owensensis* were applied to deconstruct lignocellulosic biomasses by themselves or synergetic hydrolysis with the commercial enzyme cocktail Cellic CTec2 (Novoyzmes). The aims are to comprehensively understand the lignocellulolytic enzymes of *C. owensensis* and develop new enzyme cocktails and enzymatic hydrolysis processes for bioconversion of lignocellulosic biomass.

## Results and discussion

### Growth and xylanase secretion lines

**Fig. 1:**
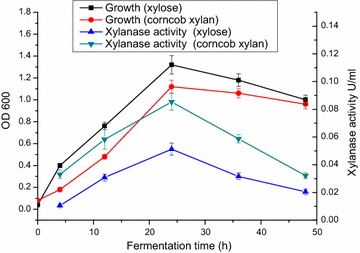
Growth and xylanase secretion on corncob xylan and xylose

Selecting a suitable incubation time for *C. owensensis* is important for assaying its enzymes. The growth and xylanase secretion lines were therefore analyzed at the beginning of this work. Figure 1 shows that regardless of xylose or corncob xylan as carbon source the cell quantity reached the highest after 24 h cultivation. The xylanase activities in the culture supernatant also increased to the peak after 24 h cultivation. The cell quantity by xylose was higher than that by xylan while the xylanase activity was reverse. It seems that xylose is more benefit for biomass accumulation while xylan can induce a higher xylanase secretion. The enzymes produced after 24 h cultivation were used in the following experiments.

### Characteristic of cellulase and hemicellulase from *C. owensensis*

The culture supernatants and cells were separated by centrifugation after 24 h cultivation. The extra-enzyme and intra-enzyme *C. owensensis* were respectively obtained after precipitation of the protein in supernatant by ammonia sulfate and breaking the cell wall by sonication. The hemicellulase (xylanase/Xyan, beta-xylosidase/pNPX, arabinofuranosidase/pNPAF, xylan esterase/pNPAC) and cellulase (endoglucanase/CMC, cellobiohydrolase/pNPC, beta-glucosidase/pNPG, filter paper activity/FPA) activities were measured.

Table 1 shows that no matter cultivated on xylose or corncob xylan both the extra-enzyme and intra-enzyme of *C. owensensis* had hemicellulase and cellulase activities. Hemicellulase and cellulase are not essential for cell growth and usually considered as the induced enzymes. However, the result of this experiment indicates that substrate inducing is not necessary for *C. owensensis* on hemicellulase and cellulase secretion. Maybe some genes for such enzymes share the promoters of the constitutive enzyme and are expressed during cell growth. On the whole, the hemicellulase activities cultivated on corncob xylan were higher than those on xylose. For example, the xylanase activities of extra-enzyme and intra-enzyme on corncob xylan were, respectively, 4.72 and 1.57 U/mg, while those on xylose were, respectively, 1.93 and 0.19 U/mg. However, the cellulase activities of the enzymes on corncob xylan and on xylose were varied slightly. This is possibly because both xylose and xylan are not the inducing substrates for cellulase secretion.

**Table 1:** Hemicellulase and cellulase activities (U/mg or mU/mg) of the crude proteins from *C. owensensis* and the thermophilic fungi reported

Microorganism and substrate	Hemicellulase activity (U/mg)				Cellulase activity (mU/mg)				References
Hydrolysis substrate	Xyan	pNPX	pNPAF	pNPAC	CMC	pNPC	pNPG	FP	
*C. owensensis* on corncob xylan									This work
Extra-enzyme	4.72 ± 0.65	0.77 ± 0.01	0.12 ± 0.01	0.27 ± 0.01	96.9 ± 7.2	25.1 ± 1.9	4.2 ± 0.3	10.7 ± 1.3	
Intra-enzyme	1.57 ± 0.074	3.14 ± 0.23	1.54 ± 0.07	0.73 ± 0.04	10.1 ± 0.8	13.0 ± 1.1	532.4 ± 37.3	5.8 ± 0.5	
*C. owensensis* on xylose									
Extra-enzyme	1.93 ± 0.15	0.04 ± 0.002	0.10 ± 0.006	0.29 ± 0.02	83.5 ± 5.8	7.8 ± 0.53	7.8 ± 0.4	6.4 ± 1.3	
Intra-enzyme	0.19 ± 0.014	0.22 ± 0.018	0.15 ± 0.008	0.24 ± 0.02	22.6 ± 1.6	44.6 ± 5.1	134.4 ± 10.6	5.3 ± 0.5	
*Thermoascus aurantiacus* on switchgrass or microcrystalline cellulose (extracellular proteins)	0.1–1.9	0.007–0.012	–	–	20–1940	9.8–34.4	45.6–177.3	–	[34]
*Thielavia terrestris* on switchgrass or microcrystalline cellulose (extracellular proteins)	0.66–3.27	0.001–0.006	–	–	230–1240	3.7–16.2	5.7–26.1	–	[34]

– no data

Table 1 also shows that the activities of extra-enzyme and intra-enzyme were different. The extra-enzyme had higher xylanase and endoglucanase activities, while the intra-enzyme had higher β-d-xylosidase, β-d-glucosidase and arabinofuranosidase activities. Especially, the β-d-glucosidase activity of intra-enzyme on corncob xylan was about 125-fold higher than that of the extra-enzyme (532.4 mU/mg VS 4.2 mU/mg). This indicates that for degrading the lignocellulosic biomass by *C. owensensis* the main function of the extra-enzyme is to cleave the polysaccharides to oligosaccharides, while further hydrolysis of xylobiose and cellobiose takes place mainly in cell by intra-enzyme.

Comparing with the thermophilic fungi *Thermoascus aurantiacus* and *Thielavia terrestris* which were recently reported to be high cellulase producers [34], the hemicellulase activities of *C. owensensis* were higher than those of fungi (Table 1, the highest activities of each enzyme were used as the results for discussing). With regard to the cellulase, it showed that although the endoglucanase activity of *C. owensensis* was much lower than those of fungi, the cellobiohydrolase activity was almost the same as those of the fungi and the β-d-glucosidase activity was much higher than those of fungi. Three enzymes, endoglucanases, exoglucanases and β-d-glucosidases, compose the cellulase system functions in a coordinated manner for degradation of cellulose into glucose units [35]. Since most glucanases are inhibited by cellobiose and short cellooligosaccharides, β-d-glucosidases catalyze the rate limiting step of the cellulose hydrolysis process as a whole [36]. Filamentous fungi are the major source of commercial cellulases. Commercial cellulase preparations are mainly based on mutant strains of *T. reesei* which have usually been characterized by a low secretion of β-glucosidase [37]. Thus, *T. reesei* cellulase preparations had to be supplemented with added β-glucosidase to provide the more efficient saccharification of cellulosic substrates [32, 37]. The enzyme system of *C. owensensis* with high hemicellulases and β-d-glucosidase activities may complement with the fungi cellulase for deconstruction of native lignocellulose.

To identify the protein components in extra- and intra-enzymes of *C. owensensis* cultivated on corncob xylan, the proteins were analyzed with HPLC/MS. More than 100 and 150 kinds of proteins were identified in the extra- and intra-enzymes respectively. Enzymes related to polysaccharide degradation were shown in Table 2. The extra-enzymes include β-xylanase (Calow_0121, Calow_1924), β-galactosidase (Calow_2098), α-N-arabinofuranosidase (Calow_0926), polysaccharide deacetylase (Calow_2141), pectate disaccharide-lyase (Calow_2109), esterase (Calow_1899), α-l-fucosidase (Calow_1765), α-amylase (Calow_0101 and Calow_0294), pullulanase, type I (Calow_0483) and a glycoside hydrolase belong to family 28 (Calow_2114). The intra-enzymes include the glycoside hydrolases belong to family 18 (Calow _2166), family 31 (Calow_1739), family 43 (Calow_2016, Calow_0925), family 4 (Calow_1700) and family 20 (Calow_0048), pullulanase, type I (Calow_0282, Calow_0483), arabinogalactan endo-β-1,4-galactanase (Calow_0481) and α-l-fucosidase (Calow_1754). Although the cellulase were not identified, the enzymes belong to different GH families might have multi-activity, including cellulolytic activity. Besides these enzymes related to polysaccharide degradation, the extra-enzymes related to carbohydrate metabolism were also identified and shown in Additional file 1, including glycosyltransferase, extracellular solute-binding protein, ATP-binding cassette (ABC) transporter-related protein, and S-layer domain-containing protein. They gave useful information for further research on carbohydrate hydrolysis and metabolism of *C. owensensis*.

**Table 2:** Detected enzymes relation to polysaccharide degradation by HPLC/MS

Entry name	Detected sequence	Gene name	Enzyme	MW kDa	Signal peptide (aa)^a^	Transmembrane domain (aa)^b^
Extra-enzymes cultivated on corncob xylan						
E4Q6K1_CALOW	E.PVVIEF.L	Calow_2098	β-galactosidase	118.37	No	No
E4Q2A1_CALOW	G.VGGNNHHQ.L	Calow_0121	β-xylanase	152.48	No	13–35
E4Q5G9_CALOW	Q.AYEGSY.S	Calow_1924	β-xylanase	187.02	1–33	5–27
E4Q4M1_CALOW	E.DAILVGCM.L	Calow_0926	α-N-arabinofuranosidase	57.89	No	No
E4Q281_CALOW	P.EIAKLY.V	Calow_0101	α-amylase catalytic region	66.17	No	No
E4Q359_CALOW	G.YDPHDYYDLGQ.Y	Calow_0294	α-amylase catalytic region	53.67	1–11	13–35
E4Q4A4_CALOW	L.VAPISMFVAYKSD.E	Calow_0483	Pullulanase, type I	127.94	No	13–32
E4Q6L6_CALOW	G.VRISNC.Y	Calow_2114	Glycoside hydrolase family 28	50.05	No	No
E4Q2L6_CALOW	T.IVGGY.K	Calow_0163	Glycoside hydrolase 15-related protein	70.76	No	No
E4Q1R5_CALOW	T.KFALPIIL.S	Calow_2141	Polysaccharide deacetylase	29.98	No	No
E4Q4M2_CALOW	I.QVISALF.E	Calow_1765	α-l-fucosidase	86.88	No	No
E4Q6L2_CALOW	T.PGDSSV.F	Calow_2109	Pectate disaccharide-lyase	188.76	No	28–50
E4Q5E4_CALOW	E.NPDPVL.V	Calow_1899	Esterase/lipase-like protein	30.17	No	No
Intra-enzymes cultivated on corncob xylan						
E4Q6Z2_CALOW	T.YEEVMALVGHHLSLN.I	Calow_2166	Glycoside hydrolase family 18	87.44	1–24	5–27
E4Q4N4_CALOW	V.VLVEK.G	Calow_0544	Glycosidase-related protein	35.84	No	No
E4Q2L6_CALOW	T.IVGGY.K	Calow_0163	Glycoside hydrolase 15-related protein	70.76	No	No
E4Q4J8_CALOW	W.SHDIAGFE.S	Calow_1739	Glycoside hydrolase family 31	88.91	No	No
E4Q6K9_CALOW	I.WAPAIRYHNGRFYIY.F	Calow_2106	Glycoside hydrolase family 43	59.87	No	No
E4Q4G6_CALOW	G.TVRLYDIDFEAAKTNEV IGNKLSS.K	Calow_1700	Glycoside hydrolase family 4	52.72	No	No
E4Q6J2_CALOW	P.VVSPER.Y	Calow_0925	Glycoside hydrolase family 43	56.05	1–26	7–29
E4Q1W6_CALOW	K.NWIF.E	Calow_0048	Glycoside hydrolase, family 20	63.68	No	No
E4Q347_CALOW	N.YDEDEGF.I	Calow_0282	Pullulanase, type I	96.51	No	No
E4Q4A4_CALOW	Y.VSGTMN.D	Calow_0483	Pullulanase, type I	127.94	No	13–32
E4Q281_CALOW	I.MYKWYLALKDKGWN.S	Calow_0101	α-amylase catalytic region	66.17	No	No
E4Q4A2_CALOW	V.AKVKVANLIQNSGF.E	Calow_0481	Arabinogalactan endo-β-1,4-galactanase	90.88	No	No
E4Q4L3_CALOW	P.QWHMK.W	Calow_1754	α-l-fucosidase	56.75	No	No

^a^Signal peptides were predicted by SignalP 4.0

^b^Transmembrane domains were predicted by TMHMM server v. 2.0

### Hydrolysis of lignocellulosic biomass by enzymes of *C. owensensis*

The enzymes of *C. owensensis* were used to hydrolyze native corn stover, native corncob and steam-exploded corn stover with the loading rate of 15 mg enzyme per gram dry substrate at 70 °C for 48 h. The experiment was performed in three groups respectively using extra-enzyme, intra-enzyme and extra-enzyme mixed with intra-enzyme at the ratio of 1:1. The data in Table 3 show that the enzyme of *C. owensensis* had high ability of degrading hemicellulose. The highest conversion rates of xylan on native corn stover, native corncob and steam-exploded corn stover were respectively 14.7, 16.8 and 59.1 %. Moreover, the conversion rates of araban on native corn stover and native corncob reached 53.5 and 60.0 %, respectively. However, the enzyme of *C. owensensis* was not such perfect at degrading cellulose. As Table 3 shows the glucose can not be detected after hydrolysis of both native corncob and steam-exploded corn stover for 48 h. This may because the endoglucanase in the enzyme of *C. owensensis* was weak (shown in Table 1). Blumer–Schuette et al. [19] analyzed the core genomes, pangenomes, and individual genomes and predicted that the ancestral *Caldicellulosiruptor* was likely cellulolytic and evolved, in some cases, into species that lost the ability to degrade crystalline cellulose while maintaining the capacity to hydrolyze amorphous cellulose and hemicelluloses. The results in this experiment were in accord with the prediction.

**Table 3:** Hydrolysis rates of lignocellulosic biomass by enzymes of *C. owensensis*

Substrate	Enzymes (15 mg/g ds)	Sugar released			
		Xylose (% of xylan)	Arabinose (% of araban)	Glucose (% of glucan)	Reducing sugar (% of carbohydrate)^a^
Native corn straw	Extra-enzyme	9.6 ± 0.32	20.9 ± 0.78	3.9 ± 0.14	15.4 ± 0.56
	Extra-/Intra- = 1:1	14.7 ± 0.56	39.5 ± 1.23	3.7 ± 0.14	14.3 ± 0.67
	Intra-enzyme	7.9 ± 0.33	53.5 ± 2.81	3.1 ± 0.12	11.5 ± 0.43
Native corncob	Extra-enzyme	11.7 ± 0.47	22.9 ± 1.12	ND	14.5 ± 0.61
	Extra-/Intra- = 1:1	16.8 ± 0.48	45.7 ± 1.73	ND	13.7 ± 0.57
	Intra-enzyme	10.4 ± 0.51	60.0 ± 2.85	ND	10.9 ± 0.49
SE corn stover	Extra-enzyme	50.0 ± 2.23	ND	ND	4.6 ± 0.17
	Extra-/Intra- = 1:1	59.1 ± 2.54	ND	ND	3.8 ± 0.16
	Intra-enzyme	36.4 ± 1.67	ND	ND	2.5 ± 0.12

*ND* Not detected

^a^Carbohydrate refers to total of glucan, xylan and araban

As described in the section of characteristic of cellulase and hemicellulase, the xylanase was mainly existed in the extra-enzyme of *C. owensensis* while the β-d-xylosidase and arabinofuranosidase were mainly existed in the intra-enzyme of *C. owensensis*. The extra-enzyme and the intra-enzyme may have synergetic function for hemicellulose hydrolysis. For each substrate, extra-enzyme mixed with intra-enzyme contributed higher levels of xylose releasing than those by extra-enzyme and intra-enzyme respective hydrolysis. However, the extra-enzyme led to the highest reducing sugar releasing, indicating that the xylanase, with the function of cleaving the xylan to xylo-oligosaccharides and xylose, is the most important enzyme for xylan degradation.

**Fig. 2:**
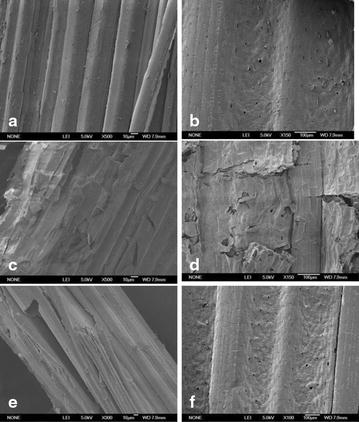
SEM of native corn stover before (**a**, **c**) and after (**b**, **d**) 48 h hydrolysis by the extra-enzyme of *C. owensensis* and the native corn stover after incubated in the acetate buffer (pH 6.0) at 70 °C for 48 h (**e**, **f**)

The morphology changes induced by hydrolysis with the extra-enzyme of *C. owensensis* were examined by SEM to provide direct insight into the structure modification in the native corn stover. Before hydrolysis, the vascular bundle and the epidermis (with stoma and epidermal hair, Fig. 2a, b) of the samples were intact. After hydrolysis, the residual corn stover was changed dramatically (Fig. 2c, d); the initial structure was destroyed and replaced by a collapsed and distorted cell wall structure. The cuticle waxy layer appeared to be almost desquamated, and the microfibrils were exposed to the surface. Clearly, the structure of the native corn stover was greatly changed in appearance after hydrolysis. Figure 2e, f shows the images of the corn stover after incubation in the acetate buffer at 70 °C for 48 h as control. When comparing with the initial corn stover (Fig. 2a, b), the acetate-buffer-incubated corn stover was slightly changed with some fissures in the sample. The acetate-buffer incubation cannot make much change for the biomass structure was proved by the hydrolysis experiment: The acetate-buffer-incubated corn stover and corncob and the samples without incubation were hydrolyzed by CTec2 (Novoyzmes) at 50 °C for 72 h. As a result, the sugar yields of the buffer-incubated samples and the corresponding samples without incubation were almost the same. The glucan conversion rates (%) were as follows: incubated corn stover 18.1 ± 1.7, corn stover without incubation 17.9 ± 1.5, incubated corncob 20.1 ± 1.6, corncob without incubation 20.4 ± 1.9.

**Fig. 3:**
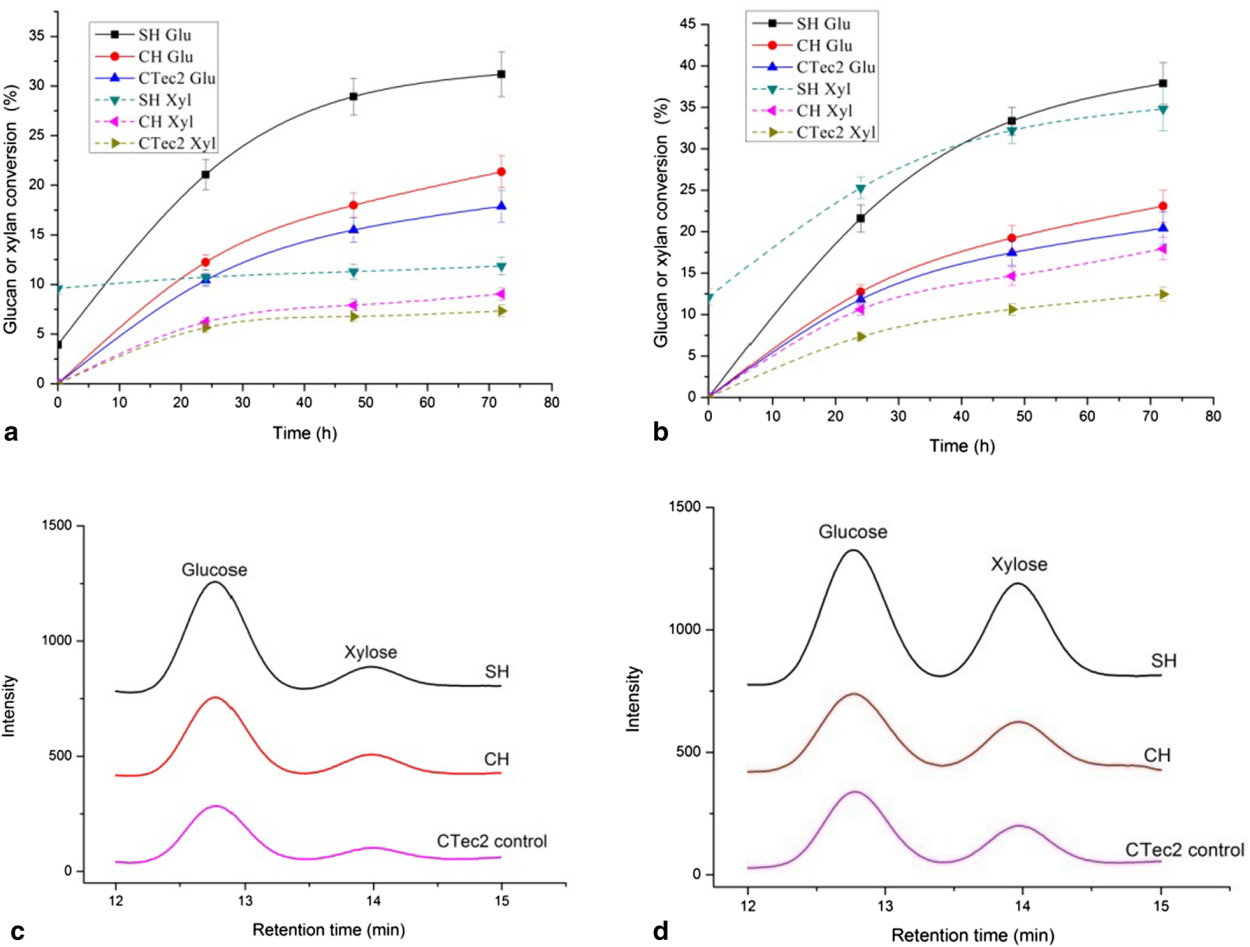
Sugar conversion rates from synergetic hydrolysis by extra-enzyme of *C. owensensis* and CTec2 on native corn stover (**a**) and native corncob (**b**). **c**, **d** are the HPLC lines of the hydrolysate at the end of hydrolysis (72 h) on native corn stover and native corncob respectively. SH (sequential hydrolysis): hydrolyzed by the enzyme of *C. owensensis* at 70 °C for 48 h (the xylan and glucan conversion rates after this hydrolysis were shown at 0 h in **a**, **b**) then adding CTec2 and incubating at 50 °C for 72 h. CH (co-hydrolysis): co-hydrolyzed by the enzyme of *C. owensensis* and CTec2 at 50 °C for 72 h. CTec2: hydrolyzed by CTec2 only as control. The loading rates of CTec2 for synergetic hydrolysis were 30 mg/g glucan (High loading). The loading rates of enzyme of *C. owensensis* for synergetic hydrolysis were 15 mg/g dry substrate. The amounts of released glucose and xylose were used for calculating glucan and xylan conversions, respectively

### Synergetic hydrolysis by the enzymes of *C. owensensis* and CTec2

Two trials were performed for synergetic hydrolysis by the enzymes of *C. owensensis* cultivated on corncob xylan and the commercial enzyme cocktail Cellic CTec2 (Novoyzmes). One was that the native corn stover and corncob were sequentially hydrolyzed (SH) by the enzymes of *C. owensensis* at 70 °C for 48 h then added CTec2 and incubated at 50 °C for 72 h. The other was that these lignocellulosic biomasses were co-hydrolyzed (CH) by the enzyme of *C. owensensis* and CTec2 at 50 °C for 72 h. The loading rates of CTec2 (http://www.bioenergy.novozymes.com/) for synergetic hydrolysis were 30 mg/g glucan (High loading). These lignocellulosic biomasses were hydrolyzed by CTec2 only at 50 °C for 72 h as controls.

**Fig. 4:**
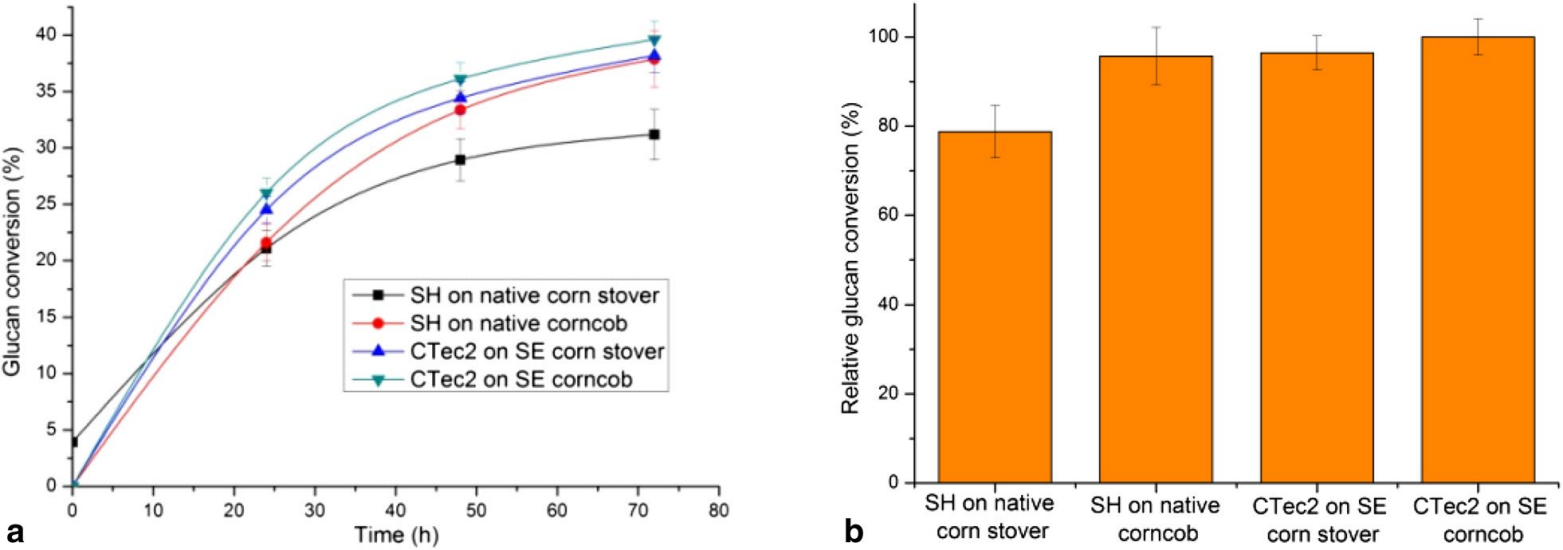
Comparison of glucan conversion rates on native corn stover and corncob by sequential hydrolysis (SH) and on steam-exploded (SE) corn stover and corncob by CTec2 alone at High loading (30 mg/g glucan). **a** Time course of glucan conversion. **b** Relative glucan conversion rate at the end of hydrolysis

Figure 3 shows that after sequential hydrolysis (SH) on native corn stover and native corncob by extra-enzyme and CTec2, the conversion rates of glucan were 31.2 and 37.9 %, which respectively were 1.7- and 1.9-fold of each control (hydrolyzed by CTec2 only). Using the same loading (high loading, 30 mg enzyme/g glucan) of CTec2 for hydrolysis of the steam-exploded (SE) corn stover and SE corncob the glucan conversion rates were 38.2 and 39.6 %, respectively (Fig. 4a), which were not much higher than glucan conversion rates of the native corn stover and corncob sequentially hydrolyzed (SH) by the enzyme of *C. owensensis* and CTec2. The glucan conversion rates of native corn stover and corncob by SH were respectively 81.7 % and 95.7 % of those of the SE corn stover and SE corncob hydrolyzed by CTec2 (Fig. 4b). It seems that in this experiment, the hydrolysis by the extra-enzyme of *C. owensensis* made almost the same contribution as steam-exploded pretreatment for glucan degradation from native lignocellulosic biomass. Sequential hydrolysis by the extra-enzyme of *C. owensensis* and CTec2 could greatly increase the hydrolysis rate for native lignocellulosic biomass possibly due to the hemicelluloses degraded by the hemicellulase in extra-enzyme of *C. owensensis* hence increasing the accessibility of cellulose to CTec2. The cellulases, especially the endoglucanase in the extra-enzyme (shown in Tables 1, 2) would also contribute to improve the cellulose hydrolysis. This is made sure by what Brunecky et al. [38] have recently reported that pre-digestion of biomass with the cellulases (CelA and endocellulase E1) from extremely thermophilic bacterium *C. becsii*, and *Acidothermus cellulolyticus* at elevated temperatures prior to addition of the commercial cellulase formulation increased conversion rates and yields when compared to commercial cellulase formulation alone.

Co-hydrolyzed (CH) by the extra-enzyme of *C. owensensis* and CTec2 the conversion rates of glucan from native corn stover and corncob were 21.4 and 23.1 % which were respectively 1.19 and 1.13 times of each control (Fig. 3). The increased extents of glucan conversion were not high as those of SH. This is because the optimum temperature for the enzymes of *C. owensensis* was 70–80 °C [33]. When co-hydrolyzed with CTec2 at 50 °C the enzyme activity was decreased.

Figure 3 also shows the xylan conversion rates from native corn stover and corncob synergetically hydrolyzed by the extra-enzyme of *C. owensensis* and CTec2. Totally, the xylan conversion rates of native corncob were higher than those of native corn stover. Especially, the xylan conversion rate of native corncob by sequential hydrolysis (SH) reached 34.8 %. The possible reason is that the enzyme of *C. owensensis* used in this experiment was produced using corncob as inducing substrate; hence, the ratio of the compositions in this enzyme was more fitted for corncob hydrolysis. It is believed that deconstructing of hemicellulose in lignocellulose will benefit cellulose degradation. This was proved by the results in Fig. 3. Namely, the higher xylose releasing (34.8 % from native corncob vs 11.8 % from native corn stover) led to a higher glucose releasing (37.9 % from native corncob vs 31.2 % from native corn stover).

**Fig. 5:**
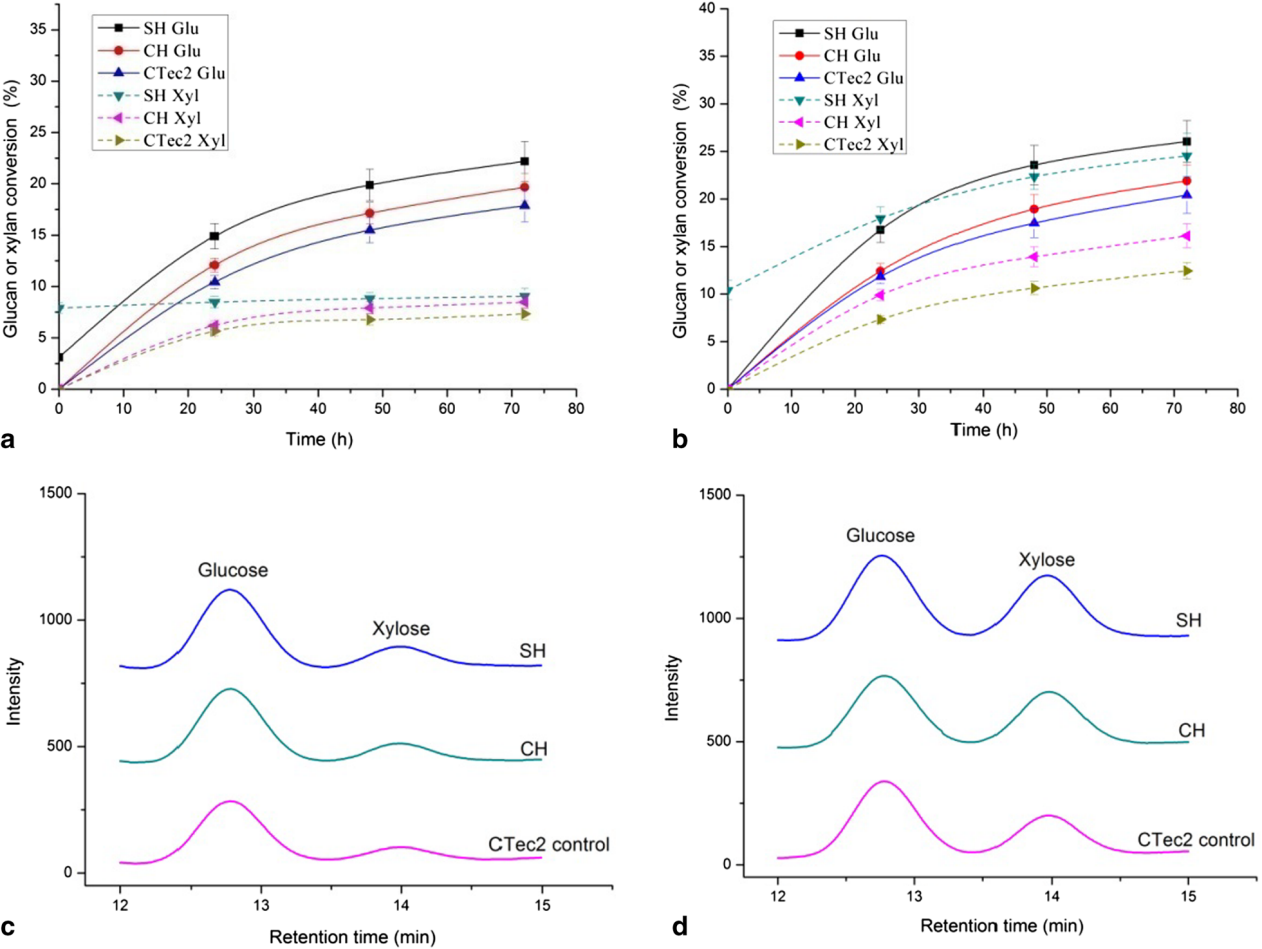
Sugar conversion rates from synergetic hydrolysis by intra-enzyme of *C. owensensis* and CTec2 on native corn stover (**a**) and native corncob (**b**). **c**, **d** are the HPLC lines of the hydrolysate at the end of hydrolysis (72 h) on native corn stover and native corncob respectively. SH (sequential hydrolysis), CH (co-hydrolysis), CTec2: hydrolyzed by CTec2 only as control. The loading rates of CTec2 and enzyme of *C. owensensis* were the same as described in Fig. 3. The amounts of released glucose and xylose were used for calculating glucan and xylan conversions, respectively

Figure 5 shows the sugar conversion rates from native corn stover and corncob by synergetic hydrolysis using intra-enzyme of *C. owensensis* and CTec2. It can be see that the conversion rates of glucan and xylan were lower than those counterparts of synergetic hydrolysis with extra-enzyme of *C. owensensis* and CTec2 (Fig. 3). This is possibly due to the higher xylanase and endoglucanase activities in the extra-enzyme.

### Inhibitors in the hydrolysate

The furfural and 5-hydroxymethyl furfural (HMF) were not detected in the hydrolysates from the native corn stover and corncob by sequential hydrolysis (SH). It is not surprising since at the temperature of 70 and 50 °C in the hydrolysis buffer the sugars are stable.

Kataeva et al. [39] found that *C. bescii* could solubilize all components of switchgrass, including lignin. Therefore, the phenolics may be released from lignin during hydrolysis by the extra-enzyme of *C. owensensis*. Really, the phenolics concentrations of the hydrolysates form native corn stover and corncob by SH were respectively 35.8 ± 3.2 and 34.3 ± 2.7 mg/l, which were slightly higher than these of the hydrolysates form native corn stover and corncob by CTec2 with 24.4 ± 1.8 and 25.1 ± 2.3 mg/l respectively. The phenolics concentrations of the hydrolysis buffer soaked (at 70 °C for 48 h) with native corn stover and corncob were, respectively, 4.2 ± 0.3 and 3.7 ± 0.3 mg/l. While the phenolics concentrations of the hydrolysates form the stream exploded corn stover and corncob by CTec2 were much high as 232 ± 17.6 and 219 ± 20.5 mg/l, respectively. These results show that only few phenolics from lignin can be released by the extra-enzyme of *C. owensensis* and CTec2. Among the biofuel-production microorganisms, *Clostridium* is very sensitive to phenolics which are lethal to *Clostridium* even at low concentrations [40, 41]. Even so, the research by Lee et al. [42] showed that the cell growth and metabolite production of *Clostridium tyrobutyricum* and *Clostridium beijerinckii* were not or slight inhibited when the phenolics concentrations were less than 100 mg/l. Wang and Chen [43] used the detoxified hydrolysate from steam-exploded rice straw to produce butanol by *Clostridium acetobutylicum* ATCC 824, and found that fermentation was improved when the phenolics concentration of the hydrolysate was less than 890 mg/l. The phenolics concentration of the hydrolysate by SH in this work was below 40 mg/l. This suggests that the hydrolysate by SH may be used for biofuels production without detoxification.

## Discussion

Pretreatment is currently an essential step for bioconversion of the lignocellulosic biomass. The common biomass pretreatment methods, such as steam-explosion pretreatment, hydrothermal pretreatment, and acid or alkali pretreatment, are costly and would inevitably lead to sugar loss and inhibitor generation [44]. The followed detoxification process is also laborious and costly. Fortunately, the extreme thermophiles *Caldicellulosiruptor* can produce a diverse set of GHs, which are different from the current commercial cellulase, for deconstruction of native lignocellulosic biomass [16–18]. Synergetic hydrolysis by these thermophilic enzymes and the commercial cellulase may efficiently deconstruct the native lignocellulosic biomass without pretreatment.

The results in this work show that the extra-enzyme of *C. owensensis* had high ability for degrading the hemicellulose of native lignocellulosic biomass. High temperature pre-hydrolysis on native lignocellulosic biomass by the extra-enzyme of *C. owensensis* could greatly improve the glucan conversion rate, making almost the same contribution as steam-exploded pretreatment which is nowadays a most widely employed method for treating the lignocellulosic biomass [7, 45, 46]. Application of the enzyme of *C. owensensis* will make it possible for avoiding the traditional pretreatment process. Based on this work, the sequential hydrolysis process by extra-enzyme of *C*. *owensensis* and commercial cellulase is presented for replacing the traditional pretreatment process for bioconversion of native lignocellulosic biomass.

**Fig. 6:**
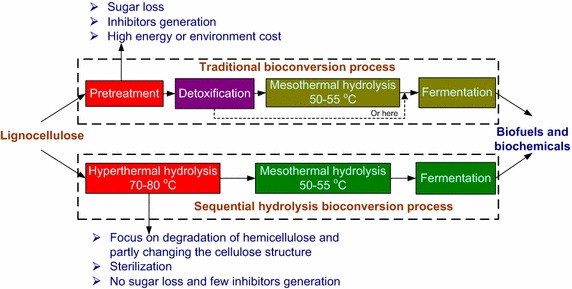
Profiles of the traditional and sequential hydrolysis bioconversion processes

The profiles of the traditional and sequential hydrolysis bioconversion processes are shown in Fig. 6. In the traditional bioconversion process, pretreatment and detoxification steps are used to improve the efficiencies of hydrolysis and fermentation. The sequential hydrolysis bioconversion process contains three sequential steps: hyperthermal enzymolysis (70–80 °C) by the extra-enzyme of *C. owensensis*, mesothermal enzymolysis (50–55 °C) by commercial cellulase and fermentation. The hyperthermal enzymolysis step, replacing the pretreatment step of traditional bioconversion process, undertakes three functions: degradation of hemicellulose, partly changing the cellulose structure and sterilization, which benefit for both mesothermal enzymolysis and fermentation. After mesothermal enzymolysis the hydrolysate may directly be used for fermentation without detoxification and sterilization. These advantages of the sequential hydrolysis bioconversion process make it a promising way for lignocellulose bioconversion.

In this work, the loading rate of the crude enzyme from *C. owensensis* was 15 mg/g dry substrate, which may not be economically feasible now. However, it could be improved by developing more efficient thermophilic hydrolase cocktails through heterogeneously expressing the relative enzymes of *C. owensensis* (shown in Table 2) and combining these enzymes in suitable composition.

## Conclusions

The extremely thermophilic bacterium *C. owensensis* has comprehensive hemicellulase and cellulase system. The enzymes of *C. owensensis* had high ability for degrading the hemicellulose of native lignocellulosic biomass. High temperature pre-hydrolysis on native lignocellulosic biomass by the extra-enzyme of *C. owensensis* could greatly improve the glucan conversion rate, making almost the same contribution as steam-exploded pretreatment. These results suggest that the costly and laborious pretreatment and detoxification steps may be removed from the bioconversion process of lignocellulosic biomass by using the enzymes from *C. owensensis*. A new process for saccharification of lignocellulosic biomass by sequential hydrolysis: hyperthermal enzymolysis (70–80 °C) by enzymes of *C. owensensis* followed with mesothermal enzymolysis (50–55 °C) by commercial cellulase is introduced in the present research. This process has the advantages of no sugar loss, few inhibitors generation and consolidated with sterilization. This research demonstrated a potential approach for bioconversion of lignocellulosic biomass without pretreatment.

## Methods

### Strain and cultivation

The extremely thermophilic cellulolytic anaerobic bacteria *Caldicellulosiruptor owensensis* DSM 13100 was purchased from the DSMZ (German Collection of Microorganisms and Cell Cultures, Braunschweig, Germany). The medium 640 (DSMZ) was used for cell cultivation and contained per liter 0.9 g NH_4_Cl, 0.9 g NaCl, 0.75 g KH_2_PO_4_, 1.5 g K_2_HPO_4_, 0.4 g MgCl_2_·6H_2_O, 2.5 mg FeCl_3_·6H_2_O, 1 ml Trace elements (medium 320, DSMZ, http://www.dsmz.de), 1 mg Resazurin, 2 g Trypticase, 1 g Yeast extract, 0.75 g Cysteine-HCl·H_2_O and 1 g xylose (or corncob xylan) as carbon source. Lyophilized cells were reactivated for 24 h at 75 °C and inoculated in new medium 640 to prepare stock cultures of 1 ml containing 30 % (w/v) glycerol which were then stored at −80 °C. The stock culture was inoculated in 10 ml of 640 medium for 16–24 h at 75 °C to be used as inoculum for growth and enzyme production tests.

Anaerobic culture media was boiled and flushed with oxygen-free N_2_ gas, distributed in Hungate tubes or serum vials and autoclaved at 121 °C for 20 min. The carbon source (corncob xylan or xylose) was added in the medium in different ways: corncob xylan was added together with other materials and autoclaved in the serum bottles with the medium, the anoxic xylose solution was filter sterilized and added with a sterile syringe to the medium after autoclave.

### Materials

The corn stover and corncob were obtained as generous gifts from a farm in Beijing suburb. The pretreatment condition [47–49] for the steam-exploded corn stover and corncob was 1.4 MPa retained for 4 min. Composition (glucan/xylan/araban/lignin content as percentage) of each lignocellulosic substrate was as follows: native corn stover (35.6/17.7/4.3/21.3), steam-exploded corn stover (45.3/2.2/0/29.6), native corncob (33.5/27.3/3.5/17.8) and steam-exploded corncob (43.7/5.5/0/23.4). The chemicals and other substrates were purchased from Sinopharm Chemical Reagent Beijing Co., Ltd or Sigma.

### Growth line assay

Bacterial cell growth was determined by measurement of the optical density (OD) at 600 nm with a spectrophotometer (Biochrom, UK). Vials containing autoclaved medium were incubated without inoculum as a control.

### Extra- and intra-enzymes extraction

After cultivation for 24 h, the culture was centrifuged at 6000×*g* for 15 min to separate the cells from the fermented broth. The cells were resuspended in a 10–15 ml binding buffer (50 mM Tris–HCl, pH 7.4, 150 mM NaCl) and broken by sonication. Then the mixture was centrifuged at 13,000×*g* for 15 min. The supernatant was collected as intra-enzymes stored in 4 °C fridge for further experiment. The protein in the cell-free fermented broth was precipitated by adding ammonia sulfate to 80 % ammonium sulfate saturation (w/v) and centrifuged at 13,000×*g* for 15 min and then resuspended in a 10–15 ml binding buffer. The binding buffer containing protein was moved into a semipermeable membrane bag and dialyzed in new binding buffer for 12 h. After this treatment the extra-enzymes were obtained.

### Protein and glycoside hydrolase assays

The protein concentrations of intra- and extra-enzymes were determined by the Coomassie Brilliant Blue G250 binding method using bovine serum albumin solution to make standard curve [50].

Endoglucanase, FPA and xylanase activities were assessed using the PHBAH (*p*-Hydroxy benzoic acid hydrazide) method using carboxymethylcellulose (CMC), filter paper (FP, Waterman) and beechwood xylan as substrate, respectively, with either glucose or xylose as the standard [51, 52]. One unit of enzyme activity was defined as the amount of protein capable of releasing 1 mmol of reducing sugar from substrate per minute. Cellobiohydrolase (pNPC, *p*-nitrophenyl-β-d-cellobioside), β-d-glucosidase (pNPG, *p*-nitrophenyl-β-d-glucopyranoside), and β-d-xylosidase (pNPX, *p*-nitrophenyl-β-d-xylopyranoside) arabinofuranosidase (pNPAF, *p*-nitrophenyl-α-l- arabinofuranoside), xylan esterase (pNPAC, *p*-nitrophenyl-acetate) activities were determined using their respective *p*-nitrophenyl sugar or ester substrates. 90 μl of sugar substrate was incubated with 10 μl of diluted enzyme, incubated for 10 min and quenched with 200 μl of 2 M sodium carbonate. The absorbance of released *p*-nitrophenol was measured at 400 nm. One unit of activities using *p*-nitrophenyl substrates was defined as the amount of protein capable of releasing 1 mmol pNP from the substrates per minute. To analyze all the enzyme activities citrate buffer (50 mM sodium citrate, pH 6.0, 150 mM NaCl) was used with the incubation temperature at 70 °C.

### HPLC/MS analysis of extra- and intra-enzymes

The enzymes were digested by trypsin. The peptides in the digest mixture were analyzed by HPLC/MS. The on-line chromatographic separation was performed by reversed-phased chromatography on an Agilent Zorbax SB C18 column (2.1 × 150 mm, 5 mm) using the Agilent 1100 system. Solvent A was water (containing 0.1 % trifluoroacetic acid), and solvent B was 60 % acetonitrile in water (v/v, containing 0.1 % trifluoroacetic acid). The gradient was that, solvent B increased from 5 to 40 % in 0–35 min, then, the solvent B increased from 40 to 50 % in 35–65 min. The flow rate was 0.2 ml/min. The outlet of the column was introduced into the ion source of an electrospray ionization mass spectrometer (LCQ DecaXP, Thermo Electron, San Jose, CA, USA). The spray voltage was set to 4.5 kV, and the heat capillary was kept at 300 °C. The data acquisition consists of three scan events, an MS scan followed by one zoomscan to determine the charge state of the ion, and MS/MS scan to provide an MS/MS spectrum. The MS scan range was set from *m*/*z* 300–2000. The zoomscan and tandem mass spectrometry (MS/MS) functions were performed in data-dependent mode. Dynamic exclusion was enabled with one count and a 0.5 min exclusion duration unit. The collision energy value was set as 35 %.

Sequence information from MS/MS data was processed using Turbo SEQUEST algorithm in Bioworks 3.2 software (ThermoElectron, San Jose, CA, USA). The protein file was created by extracting *C. owensensis* protein entries from the UniProtKB/Swiss-Prot database (http://www.uniprot.org). The peptide sequences (as shown in Table 2) were further verified based on the following criteria: (1) continuity of the b and y ion series, (2) the quality of the MS/MS spectrum, and (3) the number of ions in the MS/MS spectrum [53].

### Biomass hydrolysis and analysis

For hydrolysis by the enzyme of *C. owensensis* alone, each reaction system was prepared in 50 mM sodium acetate, pH 6.0 with the dry substrate of 2 % (w/v) and the enzyme loading of 15 mg protein per gram dry substrate. The reaction volume was 500 μl in a 2 ml Eppendorf tube, which was sealed by winding parafilm after closing the lip, and put in a water bath at 70 °C for 48 h.

For synergetic hydrolysis by the enzyme of *C. owensensis* and the commercial enzyme cocktail Cellic CTec2 (Novoyzmes), two trials were performed. One was that the lignocellulosic biomass (native corn stover or native corncob) was sequentially hydrolyzed (SH) by the enzyme of *C. owensensis* (the first step) and CTec2 (the second step). The first step was the same as described above (for hydrolysis by the enzyme of *C. owensensis* only). After 48 h hydrolysis by the enzyme of *C. owensensis* (the first step) the CTec2 and 500 μl of sodium acetate buffer, pH 5.0 were added, forming a reaction system of pH 5.0 with the dry substrate of 1 %, and then incubated in water bath at 50 °C for 72 h (the second step). The other was that the lignocellulosic biomass was co-hydrolyzed (CH) by the enzyme of *C. owensensis* and CTec2 in the sodium acetate buffer of pH 5.0 with the dry substrate of 1 % at 50 °C for 72 h. The loading rates of CTec2 (http://www.bioenergy.novozymes.com/) for synergetic hydrolysis were 30 mg/g glucan (high loading). These lignocellulosic biomasses were hydrolyzed by CTec2 alone in the sodium acetate buffer of pH 5.0 with the dry substrate of 1 % at 50 °C for 72 h as controls.

Reducing sugar assay was carried out by PHBAH method with xylose as the standard [51, 52]. Glucose and xylose concentrations were measured on a HPLC system equipped with a Hi-Plex Ca column (7.7 × 300 mm, Agilent Technology, USA), LC-20AT pump (Shimadzu, Japan) and RID-10A refractive index detector (Shimadzu, Japan), using water at a flow rate of 0.6 ml/min as mobile phase. The amounts of released glucose and xylose were used for calculating glucan and xylan conversions, respectively. The furfural and 5-hydroxymethyl furfural (HMF) were analyzed by HPLC as described above. The phenolics in the hydrolysate were analyzed by ultraviolet spectra at 280 nm using *p*-hydroxy benzaldehyde as the standard.

The native corn stover morphologies before and after hydrolysis and after incubated in the acetate buffer (pH 6.0) at 70 °C for 48 h were examined by scanning electron microscopy (SEM). The specimens were mounted on stubs and sputter-coated with gold prior to imaging with a JEOL JSM-6700F scanning electron microscope using 5-kV accelerating voltage and 10-mm distance.

## Additional file

Additional file 1: The identified extra-enzymes of *C. owensensi*s related to carbohydrate metabolism.

## Abbreviations

LCC: lignin-carbohydrate complexes
FPA: filter paper activity
CMC: carboxymethylcellulose
pNPC: *p*-nitrophenyl β-d-cellobioside
pNPG: *p*-nitrophenyl β-d-glucopyranoside
pNPX: *p*-nitrophenyl β-d-xylopyranoside
pNPAF: *p*-nitrophenyl α-l-arabinofuranoside
pNPAC: *p*-nitrophenyl acetate
SH: sequential hydrolysis
CH: co-hydrolysis
PHBAH: *p*-hydroxy benzoic acid hydrazide
HPLC/MS: high performance liquid chromatography/mass spectrometry
SEM: scanning electron microscopy
SE: steam-exploded
HMF: 5-hydroxymethyl furfural
