# Supplementary material for: Characterization of hemicellulase and cellulase from the extremely thermophilic bacterium Caldicellulosiruptor owensensis and their potential application for bioconversion of lignocellulosic biomass without pretreatment
Source: Biotechnol Biofuels. 2015 Aug 28;8:131. doi: 10.1186/s13068-015-0313-0 (PMC4552416; doi:10.1186/s13068-015-0313-0)
Supplement: Additional file 1: — The identified extra-enzymes of C. owensensis related to carbohydrate metabolism. [file 13068_2015_313_MOESM1_ESM.pdf]

Additional file 1

The identified extra-enzymes of *C. owensensis* related to carbohydrate metabolism

| Entry name | Detected sequence | m/z | Gene name |
|---|---|---|---|
| **Glycosyl transferase** | | | |
| E4Q270_CALOW | D.LPVDI.K | 556.675 | Calow_0088 |
| E4Q1U7_CALOW | G.LPVVE.A | 556.675 | Calow_0026 |
| E4Q3L8_CALOW | D.IKDLGYAIEI.I | 1135.335 | Calow_1552 |
| E4Q591_CALOW | S.ALPKFIVQ.I | 916.143 | Calow_0682 |
| E4Q2H7_CALOW | A.KTTKDILMEVYDIE.E | 1698.96 | Calow_1367 |
| E4Q1U5_CALOW | L.CVVDCRC.I | 797.992 | Calow_0024 |
| **Extracellular solute-binding protein** | | | |
| E4Q411_CALOW | R.AGVDIIIKEAQK.Q | 1285.516 | Calow_0462 |
| E4Q5R2_CALOW | A.LIGAAGD.D | 616.687 | Calow_1945 |
| E4Q5W9_CALOW | G.TIDAAR.F | 646.717 | Calow_2011 |
| E4Q4N3_CALOW | M.SILDKF.G | 722.853 | Calow_1777 |
| E4Q2A0_CALOW | A.PKSGKP.T | 613.73 | Calow_0120 |
| E4Q421_CALOW | S.KDGLTYT.F | 797.877 | Calow_1629 |
| E4Q498_CALOW | Y.PPDAAGF.W | 674.725 | Calow_0476 |
| **ABC transporter related protein** | | | |
| E4Q711_CALOW | Y.NLSGKVL.K | 730.876 | Calow_2185 |
| E4Q712_CALOW | F.LVGGY.T | 508.59 | Calow_2186 |
| E4Q3F3_CALOW | L.GIFGLLGAG.K | 804.957 | Calow_0315 |
| E4Q3C1_CALOW | I.FGIAGVDG.N | 735.808 | Calow_1524 |
| E4Q2B6_CALOW | L.FILVPRAAVSAGR.I | 1357.63 | Calow_0136 |
| **S-layer domain-containing protein** | | | |
| E4Q697_CALOW | N.LKYNILSTNRIKE.V | 1592.865 | Calow_2071 |
| E4Q696_CALOW | A.MFDLPTFTKTGE.V | 1387.584 | Calow_2070 |
| E4Q337_CALOW | F.YPSIL.L | 592.707 | Calow_0272 |
